# An adaptive classification model for peptide identification

**DOI:** 10.1186/1471-2164-16-S11-S1

**Published:** 2015-11-10

**Authors:** Xijun Liang, Zhonghang Xia, Ling Jian, Xinnan Niu, Andrew Link

**Affiliations:** 1 College of Science, China University of Petroleum, 66 Changjiang West Road, 266580 Qingdao, China; 2 Department of Computer Science, Western Kentucky University, 1906 College Heights Blvd, 42101 Bowling Green, USA; 3 Dept. of Pathology, Microbiology and Immunology, Vanderbilt University School of Medicine, 37232 Nashville, USA

**Keywords:** Peptide identification, peptide spectrum matches (PSMs), classification, support vector machine

## Abstract

**Background:**

Peptide sequence assignment is the central task in protein identification with MS/MS-based strategies. Although a number of post-database search algorithms for filtering target peptide spectrum matches (PSMs) have been developed, the discrepancy among the output PSMs is usually significant, remaining a few disputable PSMs. Current studies show that a number of target PSMs which are close to decoy PSMs can hardly be separated from those decoys by only using the discrimination function.

**Results:**

In this paper, we assign each target PSM a weight showing its possibility of being correct. We employ a SVM-based learning model to search the optimal weight for each target PSM and develop a new score system, CRanker, to rank all target PSMs. Due to the large PSM datasets generated in routine database searches, we use the Cholesky factorization technique for storing a kernel matrix to reduce the memory requirement.

**Conclusions:**

Compared with PeptideProphet and Percolator, CRanker has identified more PSMs under similar false discover rates over different datasets. CRanker has shown consistent performance on different test sets, validated the reasonability the proposed model.

## Background

As the protein plays central roles in the interaction processes, identification and quantification of proteins in a variety of samples becomes a fundamental task in proteomics [1]. In the commonly used protein identification process, mass spectrometry (MS)-based strategies coupled with sequence database searching routinely generate a large number of peptide spectrum matches (PSMs), however, only a fraction of PSMs with high confidence scores are selected as true PSMs by using statistical and machine learning algorithms [2].

For peptide identification, a number of commercial and non-commercial database search tools [3-6] have been developed to rank the PSMs based on scoring functions and report top-scored ones as target PSMs. In the early stage, empirical filters [7,8] were described to validate the target PSMs, in which all above the defined thresholds are accepted as correct and those below the thresholds are assumed to be incorrect. However, the criteria for empirical filters may not be easily defined as scoring metrics used in database search tools, the quality of the mass spectrometry data, and the type of mass spectrometer used in the LC/MS/MS experiments vary.

Recently, machine learning approaches were introduced for improving the accuracy of discrimination between correct and incorrect PSMs based on PSM data models. A widely used algorithm, PeptideProphet [9], employs an unsupervised learning approach to identify correct and incorrect PSMs. In PeptideProphet, posterior probabilities of the PSMs are computed by using the expectation maximization (EM) method based on the assumption that these PSM data are drawn from a mixture distribution of correct and incorrect PSMs. Semi-supervised learning approaches exploit decoy data and use them as references for better estimation of discriminant scores. In [10], the PeptideProphet algorithm was extended to incorporate decoy PSMs into a mixture probabilistic model at the estimation step of the EM with a semi-supervised learning framework. The restrictive parametric assumptions were removed by using the variable component mixture model and the semi-parametric mixture model. Percolator [11] is another advanced post-database searching method based on semi-supervised learning. The goal of Percolator is to increase the number of correct PSMs reported under the minimal FDR or q-value. Starting with a small set of trusted correct PSMs and a set of incorrect PSMs from searching a decoy database, Percolator iteratively adjusts the learning model to fit the dataset by ranking high-confidence PSMs higher than decoy peptide matches. The peptide identification can also be solved by a supervised learning approach which first trains a classifier with labels of PSMs already known and then uses the classifier to assign labels to those unknown PSMs [12]. In [13], a fully supervised SVM method is proposed to improve the performance of Percolator. Different with other supervised learning methods using decoy databases, De-Noise [14] labels all target PSMs as "correct", but those low-scoring ones are treated as noises. The performance of a post-database search algorithm is usually evaluated by computing FDRs based on searching a target-decoy protein database [15-19].

De-Noise has shown its efficiency on eliminating incorrect target PSMs or noisy PSMs based on weights of the protease attributes. However, parameter selection is a big challenge in De-Noise. Based on the fuzzy SVM learning model, FC-Ranker [20] needs much fewer parameters and less input from the user than De-Noise does. FC-Ranker incorporates sample clustering procedure into the SVM classifier to estimate confidence on good target PSMs. Different with the traditional SVM model, in which the weight of training error is equally contributed by each data sample, FC-Ranker uses a fuzzy classification model to estimate the possibility of each target PSM being correct. The final score of each sample is determined by the combination of the value of discriminant function and fuzzy silhouette index. However, FC-Ranker does not provide an efficient method for calculating the weight of each PSM.

Similar to [20], we cast peptide identification as a binary classification problem in which "good" PSMs are labeled as "+1" and "bad" PSMs are labeled as "-1". In this paper, to overcome the weight problem of FC-Ranker, we deal with the weight of training error as a variable, and employ the primal SVM technique [21] to re-formulate the classification problem as the CRanker classification model. In order to handle large PSM datasets, we use the Cholesky factorization technique to improve memory utilization in model training. A new scoring policy is proposed to rank all PSMs, and users can select those top-scored PSMs according to FDRs. The CRanker method has been validated on a number of PSM datasets generated from the SEQUEST database search tool. Compared with benchmark post-database search algorithms PeptideProphet and Percolator, CRanker has identified more "good" PSMs at the same false discovery rates (FDRs).

## Methods

### Peptide identification and classification problem

In sequence database searching, a large number of PSMs are routinely generated, however, only a fraction of them are correct. The task of peptide identification is to choose those correct ones from database search outputs. We formulate it as a binary classification problem, in which "good" PSMs are assigned to class "correct" or "+1" and "bad" PSMs to class "incorrect" or "-1". Different with typical classification problems, the target PSMs are not trustworthy, i.e., '+1' labels (corresponding to target PSMs) are not reliable. To overcome this problem, FC-Ranker introduces weight *θ_i _*∈ [0,1] to indicate the reliability of *i*-th PSM, where 1 represents the highest confidence level and 0 the lowest confidence level. In fact, the learning model should rely more on reliable PSMs than untrustworthy ones.

Formally, the classification problem for peptide identification is described as follows. Given a set of *l *PSMs, denoted by $\Omega ={\left\{x_{i},y_{i}\right\}}_{i=1}^{l}\subseteq R^{q}\times \left\{-1,1\right\}$ (Let $\Omega ={\left\{x_{i},y_{i}\right\}}_{i=1}^{l}\subseteq R^{q}\times \left\{-1,1\right\}$ be a set of *l *PSMs), where *x_i _*∈ *R^q ^*represents its *i*-th PSM record with *q* attributes, and *y_i _*= 1 or −1 is the corresponding label indicating a target or decoy PSM. Let

$$\Omega_{+}=\left\{j|y_{j}=1\right\},\Omega_{-}=\left\{j|y_{j}=-1\right\}.$$

SVM-based classifiers have shown its advantages in peptide identification [14,20]. A typical SVM finds a discriminant function Ψ by solving

(1) $${\text{min}}_{\Psi }\overset{l}{\underset{i=1}{\sum }}\theta_{i}Loss\left(\Psi \left(x_{i}\right),y_{i}\right)+c_{1}||\Psi ||$$

where *c*_1 _> 0 is a constant, *Loss*(Ψ(*x_i_*), *y_i_*) is the loss function of (*x_i_, y_i_*), and ||Ψ|| is the norm of Ψ for regularization. In FC-Ranker, *θ_i_, i *= 1, . . . , *l *are treated as parameters and it is a challenge to determine their values.

In [20], Problem (1) is solved by the linear programming SVM model as follows

(2) $$\begin{array}{cc} \underset{a,b,\xi ,r}{\text{min}} & -r+c\sum_{i\in \Omega }\theta_{i}\xi_{i}\\ \text{s}\text{.t}. & y_{i}\left(\sum_{j=1}^{l}\alpha_{j}y_{j}k\left(x_{j},x_{i}\right)+b\right)\geq r-\xi_{i},i\in \Omega ,\\ & r\geq 0,\\ & -1\leq \alpha_{i}\leq 1,\xi_{i}\geq 0,i\in \Omega , \end{array}$$

where *α *∈ *R^l^, b *∈ *R*^1^, *ξ *= [*ξ*_1_, . . . , *ξ_l_*] ∈ *R^l^*, and *r *∈ *R^1^*. Note that in this model, *θ_i _*is a parameter, and it is not trivial to choose a good one.

### CRanker method

#### CRanker classification model

In this section, we cope with weight *θ_i _*as a variable and re-formulate Problem (1) as CRanker classification model. A new score scheme is developed for identifying correct PSMs based on CRanker solution. Note that all '−1' labels (decoy PSMs) are reliable, and hence, *θ_i _*= 1, *i *∈ Ω_−_. Moreover, we consider constraint $\underset{i\in n_{+}}{\sum }\theta_{i}\geq \bar{\theta }$, where $\bar{\theta }>0$ is a constant, to identify as many good PSMs as possible. Hence, we solve the following optimization problem:

$$\begin{array}{cc} {\text{min}}_{\Psi ,\theta } & \sum_{i=1}^{l}\theta_{i}Loss\left(\Psi \left(x_{i}\right),y_{i}\right)+c_{1}∥\Psi ∥\\ \text{s}\text{.t}\text{.} & \theta_{i}=1,i\in \Omega_{-},\\ & 0\leq \theta_{1}\leq 1,i\in \Omega_{+},\\ & {\underset{i\in }{\sum }}_{\Omega_{+}}\theta_{i}\geq \bar{\theta ,} \end{array}$$

where *c*_1 _> 0 is a constant.

Technically, we move $\underset{i\in \Omega_{+}}{\sum }\theta_{i}\geq \bar{\theta }$ to the objective function, and reformulate model (3) as

$$\begin{array}{cc} {\text{min}}_{\Psi ,\theta } & \sum_{i=1}^{l}\theta_{i}Loss\left(\Psi \left(x_{i}\right),y_{i}\right)+c_{1}∥\Psi ∥-c_{2}\sum_{i=1}^{l}\theta_{i}\\ \text{s}\text{.t}\text{.} & \theta_{i}=1,i\in \Omega_{-},\\ & 0\leq \theta_{i}\leq 1,i\in \Omega_{+}, \end{array}$$

where *c*_2 _> 0 is a constant.

By using the primal SVM technique [21], we formulate the CRanker classification model as

(5) $$\begin{array}{cc} {\text{min}}_{\beta ,\theta } & \beta^{T}K\beta +c_{1}\sum_{i=1}^{l}\theta_{i}\cdot {\left\{\text{max}\left(0,1-y_{i}K_{i}^{T}\beta \right)\right\}}^{p}-c_{2}\sum_{i=1}^{l}\theta_{i}\\ \text{s}\text{.t}\text{.} & \theta_{i}=1,i\in \Omega_{-}\\ & 0\leq \theta_{i}\leq 1,i\in \Omega_{+}. \end{array}$$

where $K={\left(K_{ij}\right)}_{i,j=1}^{l},K_{ij}=k\left(x_{i},x_{j}\right),k\left(\cdot ,\cdot \right)$ is a given kernel, *K_i _*denotes the *i*-th column of *K*. The solution of model (5) defines a discriminant function $\Psi \left(x\right)=\overset{l}{\underset{i=1}{\sum }}\beta_{i}k\left(x_{i},x\right)$

#### Choose parameters c_1 _and c_2_

Parameters *c*_1 _and *c*_2 _play a critical role in determining the value of discrimination function Ψ(*x*_i_). We aim at Ψ(*x*_i_) ≥ 0 if *x*_i _is a correct target PSM and Ψ(*x_i_*) < 0 otherwise. Notice that *y_i _*≥ 0 for target PSMs, and *y_i _*< 0 for decoys. We have *y_i_*Ψ(*x_i_*) ≥ 0 for both correct target PSMs and decoys. Particularly, for *x_i _*with weight *θ_i_*, it contributes degree of confidence −*c*_2_*θ_i _*to the value of the objective function in problem (5). Meanwhile, *x_i _*generates an empirical loss *c*_1_*θ_i_η_i _*where $\eta_{{}_{i}}=Loss\left(y_{i},\Psi \left(x_{i}\right)\right)=\text{max}{\left\{0,1-y_{i}K_{i}^{T}\beta \right\}}^{p},p\geq 1$. In order to guarantee that the objective function of problem (5) decreases a certain amount, we enforce the loss *θ_i_*(*c*_1_*η_i _*− *c*_2_) ≤ 0, which holds if and only if $0\leq \eta_{{}_{i}}\leq \frac{c_{2}}{c_{1}}$. It implies

$$y_{i}\Psi \left(x_{i}\right)=y_{i}K_{i}^{T}\beta \geq 1-{\left(\frac{c_{2}}{c_{1}}\right)}^{1/p}.$$

Hence, if parameters *c*_1 _and *c*_2 _satisfy

$$\frac{c_{2}}{c_{1}}\leq 1,$$

we have $1-{\left(\frac{c_{2}}{c_{1}}\right)}^{1/p}\geq 0$, and then *y_i_*Ψ(*x_i_*) ≥ 0.

Moreover, if we choose parameters *c*_1 _and *c*_2 _such that $\frac{c_{2}}{c_{1}}>1$, then there exists a degeneration risk that *β *= 0 and *θ_i _*= 1 for all *i *∈ Ω_+ _(i.e., all target PSMs are identified as correct), in which case we have objective function value *l*(*c*_1 _− *c*_2_) < 0.

Therefore, we always select parameters *c*_2 _≤ *c*_1 _in CRanker.

#### Cholesky factorization for large datasets

For large PSM datasets, the kernel matrix *K *∈ *R^l×l ^*is usually not sparse, and thus, it is a big challenge to load whole *K *in memory once. Usually, the number of sample features is much less than the number of samples, and kernel function *k *provides a convenient and cheap transformation. We aim to design a low-rank approximation of large kernel matrix *K *by Cholesky factorization, and request pairwise similarities between PSMs sequentially. Specifically,

(6) $$K\approx {LL}^{T}$$

where *L *∈ *R^l,r ^, L_i,j _*= 0 if *i *<*j, L*_1,1 _≥ *L*_2,2 _≥ . . . ≥ *L_r,r _*are the square roots of the first largest *r *eigenvalues of *K*. The details can be referred to [22].

#### Calculate the scores of PSMs

Based on CRanker discriminant function Ψ(·), we assign PSM (*x_i _, y_i_*) a score

(7) $$score\left(i\right)=\frac{2}{\pi }\text{arctan}\left(\Psi \left(x_{i}\right)\right).$$

A large score value indicates the PSM is more likely to be correct. The PSMs are ordered according to their scores, and a certain number of PSMs are output to satisfy a pre-selected FDR.

## Results and discussion

We evaluated the performance of CRanker by comparing it with PeptideProphet and Percolator based on PSMs generated from the SEQUEST search engine. The CRanker algorithm was implemented with Matlab version R2010b running on a PC with Intel Core i5-2400 CPU 3.10 GHz × 4 and 8 Gb RAM.

### Experimental setup

Shotgun proteomics using multidimensional liquid chromatography coupled with tandem mass spectrometry were performed on all biological samples, including universal proteomics standard set (UPS1), the *S. cerevisiae *Gcn4 affinity-purified complex (Yeast), *S. cerevisiae *transcription complexes using the Tal08 minichromosome (Tal08) and Human Peripheral Blood Mononuclear Cells (PBMC). The RAW files generated from the different LC/MS/MS experiments were converted to mzXML format with the program ReadW. The MS/MS spectra were extracted from the mzXML file using the program MzXML2Search and all data was processed using the SEQUEST software. For PeptideProphet, we used the Trans Proteomic Pipeline V.4.0.2 (TPP), and the search outputs were converted to pep.XML format files using the TPP suite. For Percolator, we converted the SEQUEST outputs to a merged file in SQT format [23]. The UPS1 dataset, developed by Sigma-Aldrich company, contains 48 purified human proteins digested with trypsin. The SEQUEST search results include 17,335 PSMs, among which 8974 PSMs match target peptides and 8361 PSMs match decoy peptides. The Yeast dataset contains 6652 proteins and SEQUEST outputs 14,892 PSMs, among which 6703 PSMs match target peptides and 8189 PSMs match decoy peptides. For Tal08 complexes, the tryptic peptides were analyzed on an LTQ-Orbitrap XL (ThermoFisher) mass spectrometer using monoiosotopic precursor selection (MiPS). It contains 69560 PSMs, among which 42222 PSMs match target peptides and 27338 PSMs match decoy peptides. PBMCs were analyzed with both LTQ-Orbitrap XL and LTQ-Orbitrap Velos. A 6-step MuDPIT experiments was performed on a LTQ-Orbitrap XL using either MiPS (orbit-mips) or MiPS-off (orbit-nomips). The orbit-mips dataset contains 103679 PSMs, including 68334 targets and 35345 decoys, and the orbit-nomips dataset contains 117751 PSMs, including 76395 targets and 41356 decoys. For the LTQ-Orbitrap Velos experiments, 11-Step MuDPIT experiments were performed similar to Orbitrap XL experiments with either MiPS (velos-mips) or MiPS-off (velos-nomips). The velos-mips dataset contains 301879 PSMs, including 208765 targets and 93114 decoys, and the velos-nomips dataset contains 447350 PSMs, including 307549 targets and 139801 decoys. Samples are digested with trysin. There are three types of tryptic peptides: full-digested, half-digested and none-digested. The detailed PSMs are summarized in Table 1.

**Table 1:** Statistics of datasets.

		Target				Decoy			
		
	Total	Total	Full	Half	None	Total	Full	Half	None
UPS1	17335	8974	645	2013	6316	8361	236	2588	5537
Yeast	14892	6703	1453	1210	4040	8189	106	1465	6618
Tal08	69560	42222	14893	6809	20520	27338	419	5877	21042
orbit-mips	103679	68334	26760	15647	25927	35345	737	8583	26025
orbit-nomips	117751	76395	28561	17490	30344	41356	948	10333	30075
velos-mips	301879	208765	110404	35915	62446	93114	2520	24682	65912
velos-nomips	447350	307549	134117	77052	96380	139801	3414	34985	101402

Full, Half, None: number of PSMs with full-digested peptides, half-digested PSMs and none-digested PSMs, resp.

Each dataset was divided into a training set and a test set according to 50/50 ratio. For large-sized datasets, such as Tal08 and PBMCs, we randomly select 20,000 samples from the training set for model training. This procedure was repeated *n *times, and let *Ψ_i_*(x), i = 1, . . . , *n *be the discriminant function for the *i*-th time.

Then, discriminant function

$$\Psi \left(x\right)=\frac{1}{n}\overset{n}{\underset{i=1}{\sum }}\Psi_{i}\left(x\right)$$

was employed in all experiments. We set as *n *= 6 in this paper. The PSM is represented by a vector of nine attributes: xcorr, deltacn, sprank, ions, hit mass, enzN, enzC, numProt, deltacnR. The first five attributes inherit from SEQUEST and the last four attributes are defined as

• enzN: A boolean variable indicating whether the peptide is preceded by a tryptic site;

• enzC: A boolean variable indicating whether the peptide has a tryptic C-terminus;

• numProt: The number that the corresponding protein matches other PSMs;

• deltacnR: deltacn/xcorr.

Weight 1.0 was assigned for xcorr and deltacn, and 0.5 for all others. In CRanker learning model, we set parameter *c*_1 _and *c*_2 _as 1.0, *p *as 2 and choose the Gaussian kernel with kernel argument *σ *= 1.0.

## Results

Table 2 shows that the total numbers of PSMs identified by CRanker , Peptide- Prophet, and Percolator over all datasets (training and test) at *F DR *≈ 0.05. As we can see, CRanker can identify more PSMs the other two algorithms.

**Table 2:** Target PSMs output by PeptideProphet, Percolator, and CRanker.

Data	Method	Total	TP	FP
	PepProphet	582	566	16
ups1	Percolator	450	438	12
	CRanker	601	585	16

	PepProphet	1481	1443	38
yeast	Percolator	1429	1394	35
	CRanker	1491	1455	36

	PepProphet	16025	15638	387
tal08	Percolator	14725	14371	354
	CRanker	16806	16390	416

	PepProphet	34035	33233	802
orbit-mips	Percolator	34118	33270	848
	CRanker	35003	34123	880

	PepProphet	36542	35673	869
orbit-nomips	Percolator	36962	36096	866
	CRanker	37337	36416	921

	PepProphet	123908	120961	2947
velos-mips	Percolator	125701	122568	3133
	CRanker	125783	122624	3159

	PepProphet	180182	175789	4393
velos-nomips	Percolator	178082	173719	4363
	CRanker	183492	178900	4592

PepProphet: PeptideProphet. TP: number of true positive PSMs. FP: number of flase positive PSMs.

Table 3 shows the performance of CRanker on test dataset. The last column of Table 3 indicates the ratios of PSMs identified on test set and whole dataset. As the training data is randomly chosen, 50% ratio is an ideal scenario. On four PBMC datasets, the ratios are very close to 50%, indicating that CRanker classifier learned from training data works for the whole dataset. CRanker has shown very close learning performance on all datasets except UPS1. CRanker slightly overfitted on the test dataset of UPS1 (43.26%) as the training dataset is relatively small.

**Table 3:** FDR of CRanker on test set.

	TP(full/half/none)	FP(full/half/none)	FDR	$\frac{test}{total}$
ups1	253(192/57/4)	7(6/1/0)	5.38%	43.26%
yeast	730(699/30/1)	18(12/6/0)	4.81%	50.17%
tal08	8040(7299/560/181)	200(137/39/24)	4.85%	49.03%
orbit-mips	16940(13298/3370/272)	440(279/121/40)	5.06%	49.65%
orbit-nomips	18037(13918/3764/355)	459(257/144/58)	4.96%	49.63%
velos-mips	61001(54732/6006/252)	1537(1050/406/81)	4.92%	49.72%
velos-nomips	89449(66413/21364/1672)	2297(1250/937/110)	5.01%	50.00%

$\frac{test}{total}$: the ratios of PSMs identified on test set and whole dataset. FDR: false discovery rate.

**Figure 1:**
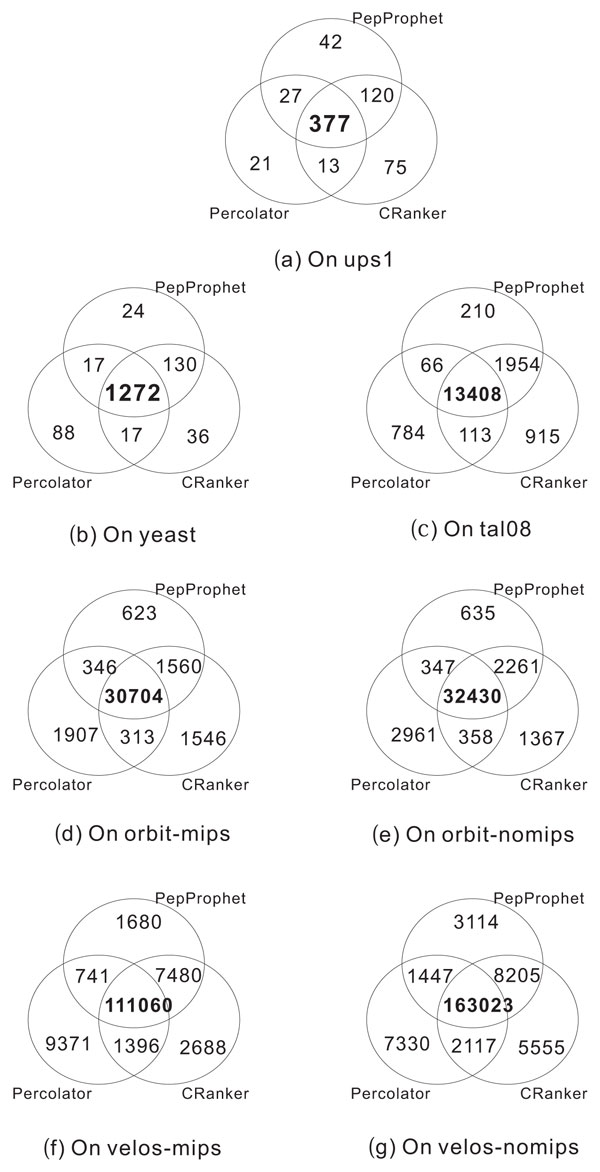
**Overlap of identified target PSMs by PeptideProphet, Percolator and CRanker**. PepProphet: PeptideProphet.

We have also looked at overlapping PSMs among PeptideProphet, Percolator and CRanker. Figure 1 shows the overlap of the identified target PSMs by the three methods on ups1, yeast, tal08 and 4 PBMC datasets. On all the datasets, the target PSMs output by CRanker have large overlap with PeptideProphet and Percolator. The details are list in Table 4. On ups1, PeptideProphet has 497 (87.8%) target PSMs shared by CRanker; Percolator has 390 (89.0%) target PSMs shared by CRanker. On all the other 6 datasets, these percentages exceed 90%. The results indicate that the majority of PSMs validated by PeptideProphet and Percolator were also validated by CRanker.

**Table 4:** Overlap of identified target PSMs by PeptideProphet, Percolator and CRanker.

	PSMs shared between Peptide-Prophet and CRanker	% Peptide- Prophet shared by CRanker	PSMs shared between Percolator and CRanker	% Percolator shared by CRanker	PSMs shared between Percolator and Peptide- Prophet	% Percolator shared by Peptide- Prophet
ups1	497	87.8	390	89.0	404	92.2
yeast	1402	97.2	1289	92.5	1272	92.5
tal08	15362	98.2	13521	94.1	13474	93.8
orbit-mips	32264	97.1	31017	93.2	30704	93.3
orbit-nomips	34691	97.3	32788	90.8	32777	90.8
velos-mips	118540	98.0	112456	91.8	111801	91.2
velos-nomips	171228	97.4	165140	95.0	164470	94.6

**Figure 2:**
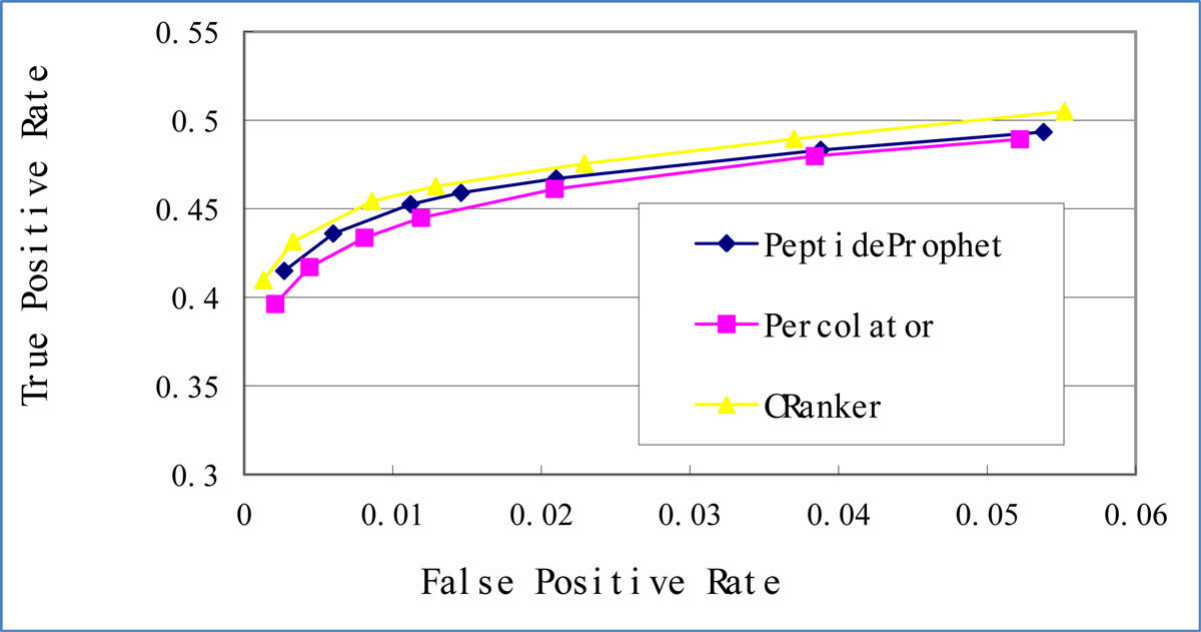
**ROC curves on orbit-nomips**.

**Figure 3:**
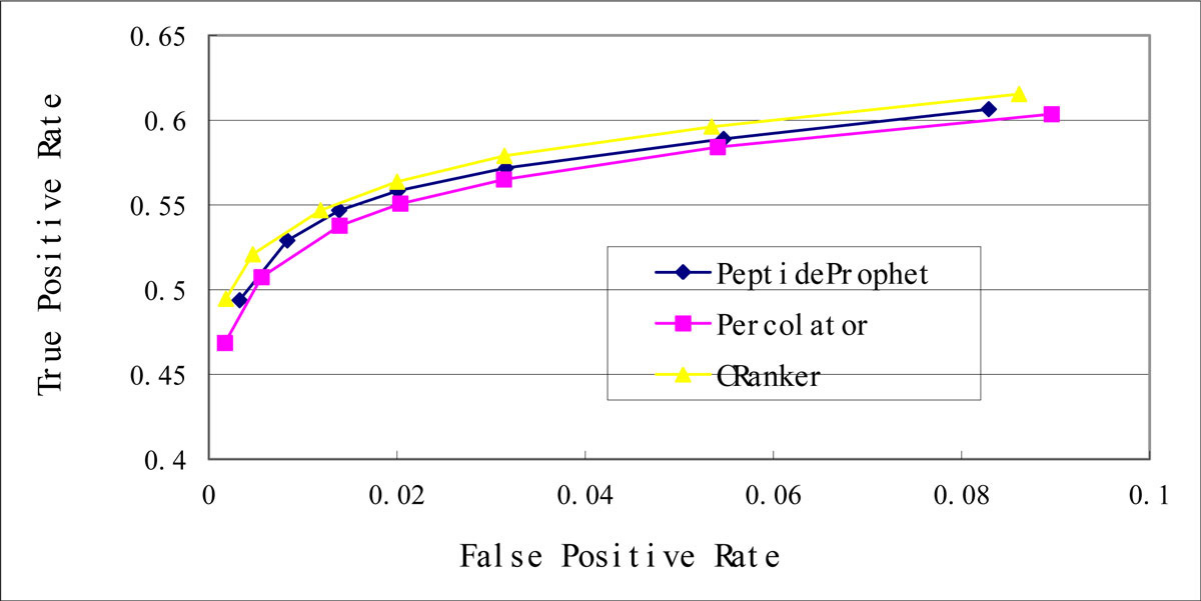
**ROC curves on velos-nomips**.

We finally compared the performance of CRanker, PeptideProphet, and Percolator by receiver operating characteristic (ROC). Due to the space limit, we included only ROCs on orbit-nomips (Figure 2) and velos-nomips (Figure 3) datasets. As we can see, CRanker reaches highest true positive rates (TPRs) throughout all false positive rates (FPRs) levels among the three algorithms in both figures.

### Stability of CRanker

As training data points are randomly chosen from training datasets, the performance of CRanker classifier may vary slightly. We counted the outputs of CRanker in 20 runs on orbit-mips and velos-mips datasets.

Let *P_i _*and #*P_i _*be the set of PSMs and the number of PSMs identified by CRanker at *i*-th run, *i *= 1, . . . , *m*. We compared the similarity of *P_i _*and *P_j _, i *≠ *j, i, j *= 1, . . . , *m *by

(8) $$s_{ij}=\frac{1}{2}\left(\frac{\#\left(P_{i}\cap P_{j}\right)}{\#P_{i}}+\frac{\#\left(P_{i}\cap P_{j}\right)}{\#P_{j}}\right).$$

Then the stability of CRanker on a dataset is defined as the mean of all pairwise similarities over *m *runs:

$$S=\frac{1}{m}\overset{m}{\underset{i,j=1,i\neq j}{\sum }}s_{i,j}$$

Table 5 and Table 6 show the numbers of PSMs identified by CRanker in 20 runs on orbit-mips and velos-mips, respectively. The stability of CRanker is *S *= 99.17% on orbit-mips and *S *= 99.53% on velos-mips.

**Table 5:** Number of PSMs identificed in 20 runs (orbit-mips).

	TP	FP		TP	FP
1	33756	882	11	33666	850
2	33772	866	12	33759	879
3	33723	854	13	33752	886
4	33756	882	14	33764	874
5	33756	882	15	33676	840
6	33558	837	16	33767	871
7	33673	844	17	33662	855
8	33680	836	18	33612	844
9	33663	853	19	33761	877
10	33747	891	20	33714	863

Average elapsed time for each run: 1848.9s.

**Table 6:** Number of PSMs identified in 20 runs (velos-mips).

	TP	FP		TP	FP
1	122272	3158	11	122286	3144
2	122252	3178	12	122266	3164
3	122241	3189	13	122286	3144
4	122250	3180	14	122268	3162
5	122267	3163	15	122246	3184
6	122278	3152	16	122005	3072
7	122009	3068	17	122031	3046
8	122289	3141	18	122033	3044
9	122001	3076	19	122034	3043
10	122284	3146	20	122034	3043

Average elapsed time for each run: 2854.5s.

## Conclusion

We have proposed a new scoring system CRanker for peptide identification, in which the confidence on each PSM is taken into account in the model training process. CRanker employs the primal SVM technique and copes with the weight of each PSM as a variable. We use the Cholesky factorization technique to improve memory utilization in model training for large PSM datasets. The performance of CRanker has been compared with benchmark algorithms PeptideProphet and Percolator over a variety of PSM datasets. The experimental studies show CRanker outperforms the other two by identifying more targets at the same FDRs.

## Abbreviations

PSM: peptide spectrum match; SVM: support vector machine; ROC: receiver operating characteristic; FDR: false discovery rate.

## Competing interests

The authors declare that they have no competing interests.

## Authors' contributions

XL and ZX designed the CRanker classification model and wrote the manuscript. LJ and XL designed the parameter selection and experiments. XN and AL provided the proteomics data and verified the experimental results. All authors read and approved the final manuscript.

## Declarations

Publication of this article was funded by the Natural Science Foundation of Shandong Province under Grant ZR2014AP004.

This article has been published as part of *BMC Genomics *Volume 16 Supplement 11, 2015: Selected articles from the Fourth IEEE International Conference on Computational Advances in Bio and medical Sciences (ICCABS 2014): Genomics. The full contents of the supplement are available online at http://www.biomedcentral.com/bmcgenomics/supplements/16/S11.
